# Workplace stress, support and stress management strategies for healthier lifestyles among healthcare workers in Ethiopia

**DOI:** 10.1371/journal.pone.0341226

**Published:** 2026-01-29

**Authors:** Zewdie Birhanu, Habtamu Mekonen, Reginald A. Kavishe, Lars Lien, Lena Skovgaard Andersen, Declare L. Mushi, Tania Aase Dræbel, Gudina Terefe Tucho

**Affiliations:** 1 Department of Health, Behavior and Society, Faculty of Public Health, Jimma University, Jimma, Ethiopia; 2 Department of Psychology, College of Education and Behavioral Sciences, Jimma University, Jimma, Ethiopia; 3 The Unisa Centre of Excellence in Disability, University of South Africa, Pretoria, South Africa; 4 School of Public Health, KCMC University, Moshi, Tanzania; 5 Faculty of Social and Health Sciences, University of Inland Norway and Innlandet Hospital, Norway; 6 Global Health Section, Department of Public Health, Copenhagen University, Denmark; 7 Department of Environmental Health Sciences and Technology, Faculty of Public Health, Jimma University, Jimma, Ethiopia; Kwame Nkrumah University of Science and Technology, GHANA

## Abstract

**Introduction:**

Workplace stress is an ongoing global and local challenge in healthcare, driven by high demands, long hours, and emotional strain, requiring urgent attention to safeguard workers’ well-being and ensure quality care.

**Objective:**

This study assessed workplace stress, support systems, and coping strategies among healthcare professionals in Jimma Zone, Ethiopia

**Methods:**

A facility based cross sectional study was conducted in Jimma Zone, Ethiopia, in August 2023. A total of 496 healthcare professionals from five different hospitals were randomly selected to participate in the study. Various validated scales, including the Workplace Stress Scale (WSS), Workplace Support for Health Scale (WSHS), and Oslo Social Support Scale (OSSS-3) were used to assess the participants’ stress levels, social support systems, and their utilization of coping strategies. The data analysis involved descriptive statistics and ordinal logistic regression techniques.

**Results:**

Results showed that 53% of healthcare workers experienced stress, with 25% reporting low, 16% moderate, and 12% severe stress. Positive workplace support for a healthier life was reported by 54% of respondents. Stress levels were significantly linked to factors like qualifications, facility type, and salary level (p < 0.05). Female workers had slightly lower stress odds than males. Workers aged 30–39 and 50–59 had lower odds of stress compared to those under 30. Higher income and working in primary hospitals were associated with reduced stress odds compared to lower income and referral hospitals.

**Conclusions:**

This study found considerable workplace stress driven by individual, organizational, and external factors, calling for targeted interventions like stress management training and resilience building programs to enhance health workers’ coping skill and improve healthcare environments in Ethiopia.

## Background

Workplace stress refers to the emotional, physical, and cognitive strain experienced by individuals due to job-related demands and pressures. It arises from various factors such as workload, deadlines, interpersonal conflicts, organizational changes, and job insecurity [1]. The impact of work-related stress on health outcomes is significant, imposing a considerable financial burden on society and healthcare system [2–4]. It can significantly impact an healthcare professionals physical health, mental well-being, job satisfaction, productivity, and overall quality of life [5]. Research indicates that workplace stress negatively affects physical and psychological health, leading to burnout and organizational losses. It can also contribute to serious health issues like cardiovascular diseases, tuberculosis, diabetes, anxiety, depression, and decreased academic and work performance [6,7]. Moreover, workplace could have direct impact on healthcare quality and workforce sustainability critically undermining both healthcare quality and workforce sustainability. When healthcare workers are overworked, underpaid, or lack proper support, it often leads to stress and burnout [5–7]. This not only affects their health and well-being but also reduces the quality of care they provide to patients. In many countries, including Ethiopia, challenges like staff shortages, heavy workloads, and limited resources make the situation worse. These conditions can cause experienced professionals to leave their jobs, creating a cycle where the healthcare system becomes even more strained [8–11]

Globally, workplace stress affects the health and well-being of workers, with organizations like the World Health Organization (WHO) and the International Labor Organization (ILO) reporting a significant portion of the global population experiencing stress related to work. In the dynamic and demanding field of healthcare, the well-being of health workers is of paramount importance [5]. However, healthcare professionals’ mental and physical health is often overlooked in healthcare, where the primary focus is patient care and well-being. Ethiopia, a country striving to improve its healthcare system, faces significant challenges related to workplace stress among its health workers. Healthcare professionals, including doctors, nurses, and other staff, operate in a demanding environment characterized by resource constraints, high patient loads, and long working hours [12]. The pressures of providing quality care in resource-constrained settings, long working hours, and high patient loads contribute to significant stress among health workers. These factors may contribute to elevated levels of stress and burnout, impacting their overall well-being and professional satisfaction.

Recognizing the negative impact of workplace stress on the health of individual workers and the healthcare system, there is an increasing awareness of the need for proactive measures to address this issue. Supporting healthier lifestyles in the workplace has emerged as a crucial element in promoting the well-being of healthcare workers and is considered one of the effective workplace stress management strategies [13,14]. This is particularly relevant in Ethiopia’s healthcare context, where efforts are underway to improve the overall health system and enhance the quality of care [15].

Workplace support for healthier lifestyles encompasses a range of initiatives to promote physical and mental well-being for health workers. In addition to promoting healthier lifestyles, effective stress management strategies are essential for mitigating the negative impact of workplace stress on health workers as they play a vital role in mitigating the adverse effects of workplace stress and promoting healthcare providers’ well-being [16].

Occupational stress among healthcare professionals is a global concern, with prevalence rates ranging from 27% to 87.4% worldwide [17]. In Ethiopia, reported stress levels among healthcare workers range between 37% and 68% [18], which is notably high compared to some global contexts. A systematic review in Ethiopia revealed that over half of Ethiopian health care workers experience occupational stress (≈52.5–52.9%), with 38.1% affected by burnout, with key risk factors including gender (being female), age ≤ 25 years, lower educational level, and job dissatisfaction, while burnout is more common among married professionals, less educated staff, and those without shifts. The prevalence of stress has remained stable over time, with women nearly four times more likely to be affected than men, and other factors such as marital status and work experience showing minimal association [8,18].

Several uniquely Ethiopian factors contribute to this burden, including chronic shortages of medical supplies, disproportionately high patient-to-staff ratios, limited mental health support services, and under-resourced healthcare infrastructure. Additionally, on-going socio-political instability, high inflation, and the lasting effects of the COVID-19 pandemic have further exacerbated stress levels among healthcare workers in Ethiopia.

Unlike many high-income countries, where systemic support, workplace wellness programs, and adequate staffing are more common, Ethiopia’s healthcare system operates with minimal resources, placing significant psychological demands on healthcare workers. While occupational stress is a global concern, the challenges faced by Ethiopian healthcare professionals are uniquely severe and complex, highlighting the need for targeted interventions and context-specific policy responses. Although workplace stress and mental health among healthcare workers have been studied globally, evidence from Ethiopia remains limited, particularly among frontline staff in primary hospital settings. Existing research has often overlooked the specific stressors these workers encounter, underreported their coping strategies, and rarely examined the availability of institutional support for promoting healthy lifestyles.

In low-resource settings such as Jimma, Oromia, healthcare workers are exposed to increasingly complex and demanding conditions that can exacerbate stress, adversely affecting both their own health and the quality of care they provide. Understanding the prevalence, predictors, and coping strategies of workplace stress in this context is therefore essential to inform evidence-based interventions and strengthen support systems that enhance healthcare worker well-being and health system resilience.

Therefore, this study addresses these critical gaps by assessing the prevalence of workplace stress, examining the availability of support for healthy lifestyles, identifying its key predictors and coping mechanism among frontline health workers in the Jimma Zone, Oromia Region, Ethiopia. The study generate context –specific evidence that inform targeted and evidence based interventions, inform policy development, strengthen support systems and ultimately strengthen frontline healthcare worker well-being and the resilience and effectiveness of the local health system.

## Methods

### Setting

A facility-based cross-sectional study was conducted in August 2023 among healthcare providers working in five government-owned hospitals in Jimma Zone, Oromia, Ethiopia.

The selected hospitals included two primary hospitals, two general hospitals, and one referral hospital. The primary and general hospitals were selected using a random sampling method, while the referral hospital was included purposively, as it is the only one in the zone. Jimma Zone is situated at 7° 40′ 0″ N latitude and 36° 50′ 0″ E longitude, approximately 350 km away from Addis Ababa, the capital of Ethiopia. The zone is divided into 21 administrative districts, hosting eight hospitals serving a population of 3722556 and 3920 workers. The five hospitals in the study provide comprehensive care and treatment to a wide range of patients, serving as COVID-19 quarantine and treatment centers. The primary hospitals accept referrals from health centers within their catchment area and, when necessary, refer patients to general hospitals or the referral hospital for further care.

### Population and sample size determination

The source population for this study comprised all categories of health professionals working in the five selected hospitals. From this source population, the study population consisted of randomly selected health professionals. Since the study employed cross sectional design, the required sample size was calculated using the single population proportion formula (n = Zα/2)^2^ p* (1-p)/d^2^), with the following parameters: a psychological impact prevalence estimate p of 61%, which is typical for perceived stress among healthcare workers in southern Ethiopia [19]; a 95% confidence interval; a margin of error of 4.5%; and a 10% adjustment for non-response. This calculation yielded a sample size of 496 health workers. To ensure representation from each hospital, the sample size was proportionally allocated to each selected hospital. Within each hospital, stratification was conducted based on the level of qualifications of health workers, namely general practitioners (GP), second degree holders, bachelor degree and diploma levels. The final sample was then selected through simple random sampling within each stratum to ensure a diverse and representative sample of health professionals across different qualifications and hospitals.

### Dependent variable

The dependent variable in this study is the Workplace Stress Scale (WSS), which measures health workers’ experiences of stress related to their workplace. The WSS consists of eight items rated on a five-point Likert scale, ranging from never (1) to very often (5).This tool was pretested to ensure clarity, cultural relevance, and ease of understanding within the local context. Participants were asked to rate how frequently each statement described their feelings about their current job. The scores for the eight items were summed to analyse the responses, resulting in a composite score ranging from 8 to 40. A higher composite score indicates a more incredible experience of workplace stress. The level/severity of workplace-induced stress was categorized based on the total score as follows: No stress (score = 8–15), low stress (score = 16–20), moderate stress (score = 21–25), and high or severe stress (score = 26–40) [13,20].

### Independent variables

#### Workplace Support for Health Scale (WSHS).

The WSHS, first developed by Christine M. Kava and colleagues at the University of Washington [21], was employed in this study as a concise five-item measure to assess health workers’ perceived workplace support for leading a healthier lifestyle (S1 Appendix).The tool was pretested to ensure clarity, cultural relevance, and ease of understanding within the local context. This encompassed perceived support from leadership, supervisors, co-workers, and wellness champions within the workplace. In addition to the original items, two items were included considering contextual factors. Thus, the final WSHS comprises seven statements, each rated on a 5-point Likert scale ranging from 1 (strongly disagree) to 5 (strongly agree). The scale demonstrated high reliability, with a Cronbach’s alpha of 0.8767.The composite score for the scale, ranging from 7 to 35, was derived by summing these items. A higher score on the scale indicates a greater level of workplace support for health [21,22].

### Oslo social support scale (OSSS-3)

The OSSS-3, originally developed by Odd Steffen Dalgard and colleagues at the University of Oslo, Norway, is a self-reported instrument designed to assess the level of social support [223] (S2 Appendix).It comprises three items that inquire about the number of close confidants, the sense of concern from others, and the relationship with neighbors, focusing on the accessibility of practical help [23]. This tool was adapted to the local context to ensure cultural relevance, and its clarity and comprehensibility were confirmed during the pretesting phase. The sum score from these items ranges from 3 to 14, where higher scores indicate stronger levels of social support, and lower scores signify poorer levels. The OSSS-3 sum score was categorized into three general levels of social support: Scores between 3 and 8 represent poor social support, Scores between 9 and 11 indicate moderate social support, and Scores between 12 and 14 signify strong social support. This tool measures the perceived level of social support among health workers, explicitly gauging their perception of how much they believe they can access during problems or when they need social support [24].

### Stress management assessment

Stress management strategies were assessed using a self-reported scale developed by Jayaraman T [25]. Respondents reported the strategies they used when facing stressful situations, with items rated on a five-point Likert scale ranging from 1 (strongly disagree) to 5 (strongly agree).. The items cover many stress management strategies, including effective time management, regular exercise, open and trusting relationships, relaxation techniques, prioritizing activities, maintaining balance in life, having close relationships with mentors, effectively utilizing and encouraging others, and redefining problems as opportunities [17,25,26] (S3 Appendix).The tool was pretested to ensure its clarity, cultural appropriateness, and comprehensibility in the local context. The scale demonstrated high reliability, with cronbach alpha of 0.8946. The items were summed up to produce a composite score where summary statistics were computed.

### Socio-demographic and training-related variables

The study collected data on various socio-demographic and training-related variables, including age, sex, education level, facility types/levels, religion, marital status, profession category, service years, experience working in COVID-19 response programs (Yes/No), receipt of COVID-19 training (Yes/No), COVID-19 vaccination status (Yes/No), and monthly salary.

### Data collection tool and procedures

Data were collected using a structured, interviewer-guided questionnaire developed based on a thorough review of relevant literature. The tool was designed to capture data on key study variables, including workplace stress, contributing factors, and available support systems. The data collection procedure was conducted as follows: First, from each selected hospital, a complete list of healthcare workers was obtained through the hospital administration. Using this list, participants were selected through simple random sampling with computer-generated random numbers. The names of the randomly selected healthcare workers were provided to trained data collectors for follow-up and invitation for participation. Second, the interviewers approached the selected participants, explained the purpose and procedures of the study, and obtained written informed consent prior to participation. After securing consent, interviewers offered a brief overview of the questionnaire and instructed participants to complete it individually. While the questionnaire including validated scales (WSS, WSHS, OSSS-3) was self-administered, interviewers remained available throughout to provide clarification, explain unfamiliar terms, and offer support when needed to ensure accurate and complete responses.

All data collectors held at least a bachelor’s degree, had prior experience in data collection, and were familiar with the local health system and cultural context. Before the actual data collection began, interviewers underwent comprehensive training covering the study protocol, ethical considerations, use of the data collection tool, and techniques for supporting participants without influencing their responses. Additionally, the questionnaire was pretested in a similar setting to ensure clarity, relevance, and reliability.

### Data analysis

The collected data underwent thorough checks for completeness and cleanliness before entering EpiData Manager Version 4.0.2. Subsequently, the data were exported to SPSS version 26 for analysis. Descriptive statistics, including proportions and means, were calculated to summarize the findings. An ordinal logistic regression was employed to analyses the outcome variable, workplace stress level, which was categorized into three ordered levels (low, moderate, and severe/high). This statistical method was deemed appropriate as it accounts for the ordinal nature of the dependent variable, allowing for the analysis of the relationship between multiple independent variables and an outcome with a natural rank order, without assuming equal spacing between categories. Multicollinearity check was conducted and found no evidence of multicollinearity among the independent variables. Moreover, the model goodness-of-fit test was conducted with being statistically non-significant (p > 0.05), indicating that the ordinal logistic regression model adequately fits the data. Significance level of p < 0.05 and a 95% confidence interval were used to determine statistically significant associations. The analysis results are presented in tables, figure, and narrative text to help you understand the findings comprehensively. The data supporting this analysis are presented in S4 Appendix.

### Data quality assurance

The data collection process was overseen by skilled data collectors proficient in local languages. The data collection tool underwent expert review to ensure its relevance and content validity. Before commencing actual data collection, a pre-test was conducted to assess the tool’s clarity and appropriateness within the local context, leading to necessary revisions for improved accuracy. Data collectors underwent thorough training and were closely supervised during data collection to maintain high standards. To further enhance data accuracy, we utilized Epidata software for data entry, which minimizes human error in the data entry process.

### Ethical consideration

The study was reviewed and approved by the Jimma University Institute of Health Ethics Review Committee (Ref. No. JUIH/IRB/307/23) as part of a broader project entitled “Strengthening Capacity to Manage and Cope with Pandemics in Ethiopia and Tanzania (SCCOPET).” The study objectives, procedures, potential risks, and benefits were clearly explained to each participant using an information sheet, and informed written consent was obtained from all participants before their involvement. Data collectors received training on ethical considerations, appropriate use of the data collection tool, and techniques for supporting participants without influencing their responses. Confidentiality was strictly maintained by de-identifying all responses, and securely storing data at Jimma University with access restricted to the research team. Participation was entirely voluntary, and participants were informed of their right to withdraw at any stage without any influence.

## Results

In this study, a total sample of 476 healthcare workers participated, resulting in a response rate of 95.8%. Table 1 presents the background characteristics of these participants. Analysis reveals that a significant proportion (57.1%) was male, and the majorities (59.3%) were below the age of 30. Marital status data showed that 56.7% of participants were married. Furthermore, 71.0% held a bachelor degree, and 36.4% had less than three years of service experience. Regarding COVID-19 involvement, a substantial portion (55.9%) reported actively engaging in pandemic response activities, with 46.4% receiving specific training. However, vaccination rates were lower, with only 33.3% having received at least one dose of the COVID-19 vaccine.

**Table 1:** Background characteristics of the study participants, Jimma (n = 476).

Variables	Categories	Frequency	%
Sex	Male	272	57.1
	Female	204	42.9
Age groups	20-29	277	59.3
	30-39	172	36.8
	≥40	18	3.9
Religion	Muslim	206	43.3
	Orthodox	157	33.0
	Protestant	101	21.2
	Others	12	2.5
Marital status	Single	193	40.6
	Married	270	56.7
	Divorced	13	2.7
Ever worked in COVID-19 response program	No	209	44.1
	Yes	265	55.9
Ever received training on COVID-19	Yes	221	46.4
	No	255	53.8
Ever received COVID-19 vaccine	Yes	88	33.3
	No	176	66.7
Do you believe COVID-19 remains an on-going pandemic?	Yes	335	70.4
	No	141	29.6
Educational qualifications	Diploma	69	14.5
	Degree	338	71.0
	MA/MSC	34	7.1
	MD	35	7.35
Health facility	Primary Hospitals	354	74.4
	Referral Hospital	122	25.6
Service year	<3 years	157	36.4
	4 to 6 years	121	28.1
	7 to 10 years	101	23.4
	More ten years	52	12.1

### Prevalence of workplace stress

The study findings indicate that 47% of the participants (95% CI: 41.9–50.9%) reported no stress issues based on self-reported data, while 53% were classified as experiencing some level of workplace-related stress. Among those reporting stress, 25% (95% CI: 21.5%−29.3%) experienced low levels, 16% (95% CI: 13.0%−19.7%) reported moderate levels, and 12% (95% CI: 8.9%−14.7%) reported severe levels of workplace-related stress. Detailed item-based responses to the workplace stress measure are presented in Table 2, and the level of workplace stress by common socio-demographic characteristics and COVID-19-related experience is presented in Table 3. Sex, qualifications, facility level, and salary scale were significantly associated with the level of workplace stress (p < 0.05).

**Table 2:** Workplace stress, measure among frontline health care workers, Jimma, Oromia (n = 476).

WPS items	Never	Rarely	Sometimes	Often	Very Often
Conditions at work are unpleasant or sometimes even unsafe	31.6	30.1	20.8	9.9	7.6
I feel that my job is negatively affecting my physical or emotional well-being.	48.2	19.2	18.3	9.7	4.6
I have too much work to do and/or too many unreasonable deadlines.	36.4	28.6	22.3	8.8	3.8
I find it difficult to express my opinions or feelings about my job conditions to my superiors.	40.8	24.2	18.5	10.7	5.7
I feel that job pressures interfere with my family or personal life.	42.1	24.2	19.4	9.1	5.3
I feel that I have inadequate control or input over my work duties.	48.8	18.7	19.0	8.8	4.6
I receive inadequate recognition or rewards for good performance.	35.4	21.9	20.6	11.4	10.7
I am unable to fully utilize my skills and talents at work.	49.1	19.0	19.8	7.6	4.6

**Table 3:** The level of workplace stress by common socio-demographic characteristics and COVID-19 related experience, Jimma, Ethiopia ((n = 476).

Variables	Categories	Standard (no stress)	Low	Moderate	Sever	Total	P-value
Sex	Male	111(50.5)	76 (62.8)	43 (55.1)	41 (73.2)	41 (73.2)	0.009
	Female	109 (49.6)	45 (37.2)	35 (44.9)	15 (26.8)	15 (26.8)	
Age in years	20-29	127 (59.4)	67 (56.3)	41 (53.3)	41 (73.2)	276 (59.2)	0.331
	30-39	78 (36.5)	46(38.7)	35 (45.5)	13 (23.2)	172 (36.9)	
	40-49	6 (2.8)	5 (4.2)	1(1.3)	2 (3.6)	14 (3.0)	
	50-59	3 (1.4)	1 (0.8)	0 (0.0)	0 (0.0)	4 (0,9)	
Religion	Orthodox	69 (31.4)	44 (36.4)	23(29.5)	20(35.7)	156 (32.8)	0.347
	Muslim	92(41.8)	57(47.1)	38 (48.7)	19(33.9)	206 (43.4)	
	Protestant	53 (24.1)	16 (13.2)	16(20.51)	16 (28.6)	101 (21.3)	
	Others	6 (2.7)	4 (3.3)	1 (1.3)	1 (1.8)	12 (2.53)	
Marital Status	Single	89 (40.5)	46 (38.0)	32 (41.0)	26 (46.4)	193 (40.6)	0.917
	Married	126 (57.3)	72 (59.5)	43 (55.1)	28 (50.0)	269 (56.6)	
	Divorced	5 (2.3)	3 (2.5)	3 (3.9)	2 (3.6)	13 (2.7)	
Worked in COVID program	No	99 (45.4)	47 (38.8)	38 (48.7)	25 (44.6)	209 (44.2)	0.534
	Yes	119 (54.6)	74 (61.2)	40 (51.3)	31 (55.4)	264 (55.8)	
Training COVID-19?	Yes	104 (47.3)	51 (42.2)	40 (51.3)	25 (44.6)	220 (46.3)	0.623
	No	116 (52.7)	70 (57.9)	38 (48.7)	31 (55.4)	255 (53.7)	
COVID-19 vaccine?	Yes	149 (67.7)	89 (73.6)	50 (64.1)	37 (66.1)	325 (68.4)_	0.505
	No	71 (32.3)	32 (26.5)	28 (35.9)	19 (33.9)	150 (31.6)_	
COVID-19 still pandemic?	Yes	148 (67.3)	91 (75.2)	53 (68.0)	42 (75.0)	334 (70.3)	0.367
	No	72 (32.7)	30 (24.8)	25 (32.1)	14 (25.0)	141 (29.7)	
Qualifications types	Diploma	44 (20.0)	11 (9.1)	12 (15.4)	2 (3.6)	69 (14.5)	0.001
	Degree	155 (70.5)	88 (72.7)	49 (62.8)	45 (80.4)	337 (71.0)	
	MA/MSC	14 (6.4)	7 (5.8)	6 (7.7)	7 (12.5)	34 (7.2)	
	MD	7 (3.4)	15 (12.4)	11 (14.1)	2 (3.6)	35 (7.4)	
Facility level	Referral Hospital	48 (21.8)	22 (18.2)	29 (37.2)	19 (33.9)	118 (24.8)	0.005
	Primary Hospital	172 (78.2)	99 (81.8)	49 (62.8)	37 (66.1)	357 (75.2)	
Serves year	0 to 3 years	78 (39.0)	39 (34.8)	22 (31.4)	18 (36.7)	157 (36.4)	0.582
	4 to 6 years	54 (27.0)	26 (23.2)	27 (38.6)	14 (28.6)	121 (28.1)	
	7 to 10 years	46 (23.0)	29 (25.9)	15 (21.4)	11(22.5)	101 (23.4)	
	More ten years	22 (11.0)	18 (16.1)	6 (8.6)	6 (12.2)	52 (12.1)	
Salary	<4500	56 (26.5)	50 (43.1)	28 (38.4)	24 (42.7)	158 (34.7)	0.007
	4501-7500	132 (62.6)	63 (54.3)	40 (54.8)	30 (53.6)	265 (58.1)	
	>7500	23 (10.9)	3 (2.6)	5 (6.6)	2 (3.6)	33 (7.2)	

### Prevalence of workplace support for (WPS) health lifestyle

**Fig 1:**
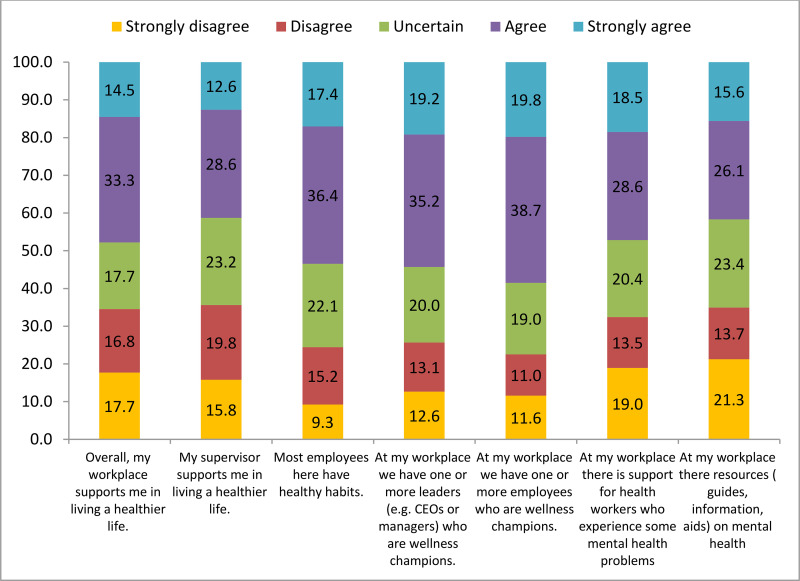
Distribution of responses to the Workplace Support (WPS) items, Jimma, Ethiopia.

In this study, we evaluated the perceived support system within workplaces for leading a healthier life and detailed the responses to specific items (Fig 1).

**Fig 1 presents the percentage distribution of responses across five Likert-scale categories for seven workplace support statements.**

Higher levels of agreement were observed for items related to healthy workplace habits and the presence of wellness champions among leaders and employees. In contrast, lower agreement and higher disagreement were reported for support for workers experiencing mental health problems and for the availability of mental health resources. Overall, combined disagreement (strongly disagree or disagree) ranged from approximately 13% to 21% across items, reflecting variability in perceived workplace and mental health support. For example, 17.7% of respondents strongly disagreed and 14.5% strongly agreed that their workplace supports them in living a healthier life. Similarly, nearly one-third of respondents expressed disagreement regarding support for health workers facing mental health challenges, and more than one-third reported disagreement with the availability of mental health–related resources at their workplace.

The average mean score for perceived workplace support was 22.4 (SD = 6.9, possible value range: 7–35), indicating a varied perception among participants. Notably, more than half (53.6%) of the respondents scored above this mean value, suggesting a generally positive outlook on workplace support. Chi-square analysis (result not shown in table) demonstrated significant difference of workplace stress in relation to some factors and perceived workplace support. Specifically, working experience in COVID-19 response (48.3% vs. 57.7%, p = 0.04) and the type of workplace setting (primary vs. referral hospitals) were significantly associated with perceived workplace support. Notably, primary hospitals showed higher positive workplace support, with a p-value of 0.001, indicating a substantial difference in perceived support between these settings. However, other factors such as age, sex, religion, and educational level did not show a significant association with perceived workplace support (P > 0.05)

### Prevalence of social support

The study found that only 14.5% of the respondents had a moderate level of social support, and none rated their social support as good based on the SSS-3 classification scale. A slightly higher proportion of females (16.2%) than males (13.2%) tended to perceive their social support as good though these associations are statically not significant. The detailed response to each item is shown in Table 4. The mean scale score was 6.8 (SD = 1.6), with a possible range of −3–14, indicating a range of perceptions regarding social support among the respondents.

**Table 4:** Perceived social support by gender based on the SSS-3 classification scale (n = 476).

SSS-items	Male (n = 272)	Female (n = 204)	Total (n = 476)	P-value
**How many people can you count on if you have severe personal problems?**				**0.230**
None	52 (19.1)	42 (20.6)	94 (19.7)	
One or two	95 (34.9)	86 (42.2)	181 (38.0)	
Three to five	69 (25.4)	38 (18.6)	107 (22.5)	
More than five	56 (20.6)	38 (18.6)	94 (19.7)	
**How much concern do people show in what you are doing?**				**0.180**
No concern or interest	105 (38.6)	90 (44.1)	195 (41.0)	
Uncertain	104 (38.2)	66 (32.4)	170 (35.7)	
Some concern and interest	52 (19.1)	33 (16.2)	85 (17.9)	
A lot of concern and interest	11 (4.0)	15 (7.4)	26 (5.5)	
**How easy is it to get practical help from neighbors?**				**0.085**
Very difficult	50 (18.4)	28 (13.7)	78 (16.4)	
Difficult	86 (31.6)	59 (28.9)	145 (30.5)	
Easy	119 (43.8)	92 (45.1)	211 (44.3)	
Very easy	17 (6.3)	25 (12.3)	42 (8.8)	
**Overall social support**				
Moderate social support	36 (13.2)	33 (16.2)	69 (14.5)	
Poor social support	236 (86.8)	171 (83.8)	407 (85.5)	

### Prevalence of self-reported stress management strategies

Many respondents (69.6%) use effective time-management methods like keeping track of time, making to-do lists, and prioritizing tasks during stressful situations. About 52.5% maintain a regular exercise program, showing a strong commitment to physical well-being as a coping strategy. Additionally, 66.7% maintain trusting relationships for sharing frustrations, emphasizing the importance of social support—furthermore, 53.6% practice relaxation techniques, indicating an active effort in stress management. Moreover, 64.5% prioritize important matters, balancing their focus effectively. While 57.4% pursue diverse interests outside work for balance, 33.7% find this balance challenging. Many (61.1%) have mentorship relationships aiding their coping mechanisms and a similar number (61.1%) collaborate effectively with others for work assignments, highlighting teamwork as a coping strategy. Encouragingly, 70.6% promote solutions over problems, showcasing proactive problem-solving in their environment. Similarly, 64.1% view problems as opportunities for improvement, displaying a positive and resilient mindset in facing challenges (Table 5). The average score was 35.6 (SD = 9.2) with a median value of 38.0 (range of possible value: 10–50), and 273 (57.4%) respondents scored above the mean value.

**Table 5:** Stress management practices among frontline health workers in Ethiopia, Jimma (n = 476).

When I faced with stressful situation	No (SD, DA, Un)	Yes (SA +Agree)
I use effective time-management methods such as keeping track of my time, making to do lists, and prioritizing tasks.	30.5%	69.6%
I maintain a program of regular exercise for fitness.	47.5%	52.5%
I maintain an open, trusting relationship with someone with whom I can share my frustrations.	32.9%	67.0%
I know and practice several temporary relaxation techniques such as deep breathing and muscle relaxation.	46.4%	53.6%
I frequently affirm my priorities so that less important things don’t drive out more important things.	35.4%	64.5%
I maintain balance in my life by pursuing a variety of interests outside of work.	42.7%	57.4%
I have a close relationship with someone who serves as my mentor or advisor.	38.9%	61.1%
I effectively utilize others in accomplishing work assignments.	38.8%	61.1%
I encourage others to generate recommended solutions, not just questions, when they come to me with problems or issues.	29.4%	70.6%
I strive to redefine problems as opportunities for improvement.	36.0%	64.0%

*Note: No = strongly disagree, Disagree and uncertain, Yes = Strongly agree and agree*

### Factors influencing workplace stress levels

Variables with significant associations during binary analysis (p < 0.05) were entered into the adjusted analysis to explore their links with workplace stress, as detailed in Table 6. Age exhibited a substantial association with workplace stress, with individuals aged 30−39 years (OR=0.59, p = 0.036) and 50−59 years (OR=0.09, p = 0.068) having lower odds of experiencing stress than those under 30. Higher income levels (> 7500 ETB and 4501−7500 ETB) were significantly associated with reduced odds of stress (OR=0.31 and OR=0.53, respectively) compared to lower income (<4500 ETB). Working in primary hospitals was significantly associated with lower odds of stress (OR=0.55) compared to referral hospitals. Likewise, experience of negative life events were positively associated with higher state of workplace stress (p = 0,001). Other socio-demographic variables such as religion, marital status, qualifications, COVID-19 response and training, COVID-19 vaccine, and service years also showed no significant associations with workplace stress. Additionally, variables related to social support, workplace health support, and stress management were not significantly associated with workplace stress (p > 0.05).

**Table 6:** Factors associated with workplace stress, Jimma, Ethiopia (n = 476).

Variable	OR	P-value	[95% CI)	
Sex (Females/Male*)	0.71	0.096	−0.76	0.06
Age category in years (<30 years*)				
30–39 years	0.59	0.036	−1.02	−0.03
40–49 years	0.48	0.243	−1.98	0.50
50–59 years	0.09	0.068	−5.00	0.18
Religion (orthodox*)				
Muslim	1.27	0.288	−0.20	0.68
Protestant	1.16	0.612	−0.41	0.70
Others	0.96	0.946	−1.34	1.25
Marital status (single*)				
Married	0.91	0.666	−0.53	0.34
Divorced	1.98	0.249	−0.48	1.84
Ever worked in COVID-19 response (Yes/No*	1.10	0.693	−0.38	0.57
Ever received training on COVID-19 (Yes/No*)	1.32	0.225	−0.17	0.73
Received COVID-19 vaccine (Yes/No*)	1.19	0.476	−0.30	0.64
Qualifications (diploma*)				
Degree	1.21	0.574	−0.47	0.84
MA/MSC	1.38	0.519	−0.66	1.31
MD	1.35	0.560	−0.72	1.32
Service years (<4 years*)				
4–6 years	1.42	0.202	−0.19	0.88
7–10 years	1.10	0.768	−0.53	0.72
More ten years	1.60	0.269	−0.36	1.31
Salary income (<4500 ETB *)				
4501–7500 ETB	0.53	0.020	−1.19	−0.10
> 7500 ETB	0.31	0.040	−2.30	−0.05
Workplace type (Referral*/Primary Hospitals)	0.55	0.024	−1.13	−0.08
Social support	1.08	0.278	−0.25	0.86
Negative live event	4.86	0.001	0.12	0.29
Workplace health support	−0.42	0.677	−0.04	0.02
Stress management	−0.25	0.800	−0.03	0.02

*reference category

## Discussion

Stress management in healthcare settings is not only essential for the well-being of healthcare workers but also has direct implications for healthcare system performance and effectiveness. Evidence suggests that effective stress management interventions can lead to improved job satisfaction, reduced burnout, and enhanced performance, which in turn contribute to better patient outcomes, lower staff turnover, and overall system efficiency [27,28]. Therefore, addressing stress among healthcare professionals is a critical component of strengthening healthcare delivery and ensuring sustainable workforce performance. In this study, we aimed to investigate the prevalence of workplace stress, the availability of workplace support for healthier lifestyles, and the use of stress management strategies among health workers in Ethiopia. The study revealed varying levels of workplace stress among health workers in the study area, with certain socio-demographic factors showing significant associations with it, with no COVID did not show significant association with workplace stress levels.

The data reveal that half of the participants experienced work-related stress. This finding is consistent with the global recognition of workplace stress as a widespread issue, particularly in high demand sectors such as healthcare [29,30]. Further analysis categorized the participants stress experience into different levels: highlighting the varying degrees of impact that workplace stress can have on individuals’ psychosocial well-being, job performance and job dissatisfaction and turnover [13]. Studies in Ethiopia and across various countries have documented high levels of workplace stress among healthcare workers, ranging from around 30% to over 57.5% [7,23,31,32] with pooled estimates from a meta-analysis of 52.5% [18], comparable with the present study. Yet, studies also reported high levels of stress, as high as 70% [13,33] in Ethiopia. Indeed, the finding that nearly half of the participants reported no stress issues based on self-reported data is noteworthy. It suggests that while a substantial portion of health workers may manage stress effectively or perceive their stress levels as manageable, a significant proportion remains facing notable stress levels. This underscores the importance of tailored interventions and support mechanisms to address stressors effectively across the health workforce spectrum, considering their experience and ability to manage and poorly manage their stress.

An essential aspect of mitigating workplace stress is the availability of workplace support mechanisms and stress management strategies. The findings underscore the critical role of workplace support in mitigating health workers’ stress, aligning with established organizational psychology frameworks. This is because the workplace support plays a crucial role in reducing stress, consistent with the Job Demands-Resources model, which identifies social support as a buffer against job stress and burnout [34]. Support from supervisors and colleagues enhance employees’ coping ability and well-being, aligning with evidence that supportive environments improve psychological health and job satisfaction [35]. Thus, promoting workplace support can benefit both employees and organizational outcomes. In light of this, our study’s results highlight various opinions among respondents regarding the support they perceive within their workplaces for leading a healthier life. The study also sheds light on specific areas of concern within workplace support for health workers. For example, a notable percentage of respondents expressed concerns that support for health workers facing mental health challenges is unavailable. This finding underscores the importance of addressing and promoting healthier lifestyle options at the workplace, comprehensively within healthcare settings, including access to resources, establishing organizational policies that can identify factors that contribute to stress at the workplace, and indicating appropriate interventions and on-going training on reducing workplace stressors.

Moreover, the findings regarding the availability of resources related to mental health at workplaces are limited. This indicates potential gaps in providing essential tools and information to create favorable conditions for stress management among health workers, which is crucial given the high prevalence of workplace stress among healthcare providers. It’s evident that the availability of workplace support for health workers’ well-being can vary widely and is influenced by various factors such as organizational culture, leadership style, the availability of resources and the organizational psychology [36]. This led to gaps in mental health support and resource availability at the workplace, which is consistent with broader discussions on the need for increased interventions and attention to healthcare workforce well-being locally. These findings call for targeted interventions and policies to improve workplace support for health workers’ well-being. This may involve implementing stress management awareness programs, training managers and colleagues to recognize and support mental health challenges, ensuring access to counselling services, and promoting a culture of open communication and support within healthcare organizations. Moreover, addressing the gaps in health workplace support for a healthier life may require implementing comprehensive wellness programs, training supervisors, promoting healthy habits organization-wide, identifying and empowering wellness champions, improving mental health support systems, and enhancing awareness and utilization of resources.

The study revealed several important insights concerning stress management skills and practices among frontline health workers, including positive and negative aspects. The studied health workers had insufficient workplace stress management skills and practices. However, while a significant proportion of respondents reported using effective time-management methods and maintaining trusting relationships, there were notable areas of concern. For instance, only half of respondents support a regular exercise program for fitness, indicating a potential gap in promoting physical well-being among frontline health workers. Similarly, many respondents do not frequently affirm their priorities (35.4%) or maintain balance in life by pursuing diverse interests outside of work (42.7%). This finding aligned with existing literature on stress management among frontline health workers in which many health workers lack effective strategies, and experience risking burnout [1], limited time-management techniques and trusting relationships [2], the limited support for exercise [3]. Furthermore, low engagement in self-affirmation and diverse interests highlights a need for improvement, as a healthy work-life balance is essential for preventing burnout [4]. This suggests areas where interventions to promote self-awareness and work-life balance could be beneficial.

Additionally, a significant percentage of respondents do not have a close relationship with a mentor or advisor (38.9%) and do not effectively utilize other colleagues in accomplishing work assignments (38.8%), highlighting the need to improve support systems and teamwork and fostering a proactive problem-solving culture within healthcare settings. These negative findings underscore the need for comprehensive stress management programs and training that reinforce existing positive practices and address areas of improvement, such as promoting physical activity, enhancing support networks, encouraging work-life balance, and fostering a proactive and collaborative approach to problem-solving. Some earlier studies highlighted that effective training program for healthcare professionals can help reduce stress and increase resilience [20]. Further research could explore the barriers and challenges frontline health workers face in adopting effective stress management strategies and tailor interventions to address these specific needs, ultimately contributing to improved well-being and job satisfaction in resource-limited healthcare settings where COVID-19 has further strained resources.

Gender differences were observed, although the difference in stress levels between males and females was not statistically significant in this study. The finding that males show a non-statistically lower odds ratio for workplace stress compared to females aligns with some literature indicating that females often report higher stress levels [13,17,29,31–33]. This could be attributed to the combined effects of societal pressures, structural gender disparities, and workplace systems that often fail to accommodate the needs of women. This suggests that gender-specific stressors and coping mechanisms should be considered in workplace stress management programs. Furthermore, age emerged as a significant factor, with older health workers showing lower odds of experiencing stress compared to their younger counterparts. The significant association between age and workplace stress, notably lower odds for individuals aged 30–39 and 50–59 years, is consistent with studies highlighting that older employees may have developed better-coping strategies or face different stressors than younger counterparts [37–41]. However, some previous studies also reported substantially higher workplace stress among older employees [42]. These findings underscore the need for age-sensitive and context-specific strategies in workplace stress management programs within the healthcare sector. By tailoring these programs to different age groups, with a special focus on helping younger employees effectively manage workplace stress, healthcare organizations can better support the well-being of their workforce.

The positive relationship between salary levels and workplace stress, with higher-income employees experiencing lower stress levels and vice versa, is in line with socio-economic stress theories [43].This is also supported by a study in Ethiopia, which stated that healthcare workers with lower salaries tended to experience more workplace stress [44]. Previous studies also reinforce the current findings in that healthcare workers in Africa face significant workplace stress and low pay relative to their workload. In Ethiopia, 68% of healthcare staff report occupational stress, with 57.6% experiencing job anxiety and 39% experiencing depression—primarily due to long hours and poor management [45]. In Rwanda, over 57% of hospital staff report burnout, with 71% experiencing fatigue, particularly among doctors, nurses, and midwives [46]. In Tanzania, 62% of acute-care workers report burnout, with emotional exhaustion being particularly high [47]. Low-income health workers may experience workplace stress due to financial challenges, job insecurity, adverse work environments, limited social support, and access to resources within and outside the healthcare environment. This underscores the need for equitable support systems and resources across income brackets to mitigate stress disparities. Moreover, workplace stress management interventions and support must design tailored strategies for health workers with different salary levels. These findings resonate with studies emphasizing the importance of equitable compensation to sustain the healthcare workforce across Africa while considering diverse socio-demographic factors in understanding and addressing workplace stress [44,48].

The significant association between lower stress levels in workers at primary hospitals compared to those at referral hospitals suggests that organizational factors play a crucial role in determining stress levels. This finding is supported by research indicating that work environment, workload, and support mechanisms significantly influence employee stress [49,50]. The absence of significant associations with COVID-19-related variables—such as response efforts, training, vaccination, and social support—may indicate a prevailing perception that the COVID-19 pandemic is perceived to be over. These factors show significant associations with workplace stress and emphasize the crucial role of support systems, resilience-building strategies, and addressing external stressors in mitigating workplace stress. These findings are consistent with literature highlighting the importance of social support and effective stress management techniques in promoting employee well-being. This is because social support had a threefold effect on work stressors–strain relations, helped to reduce the strains experienced, and mitigated perceived stressors [51,52]. Overall, this study highlights that although Ethiopian healthcare professionals experience high levels of workplace stress driven by multiple factors, there are no specific national or regional strategies or policies in place to address this issue. This gap calls for urgent attention and the development of context-specific policy guidelines to better support the health workforce.

### Strengths and limitations of the study

This study has several strengths. First, a random sampling method was employed, which enhances the representativeness of the sample. Second, validated instruments (WSS, WSHS, and OSSS-3) were utilized to ensure the reliability and credibility of measurements related to stress and coping strategies. In addition, the inclusion of multiple sites across different levels of health facilities and the participation of five hospitals strengthen both the representativeness and the generalizability of the findings within the Jimma Zone and beyond. However, the cross-sectional design limits the ability to infer causal relationships. Additionally, the use of self-reported data may have introduced social desirability bias, potentially affecting the accuracy of participants’ responses. Additionally, as data were collected in the workplace, responses may have been influenced by context/environmental bias, with interviewees altering answers under observation and interviewers affected by workplace dynamics.

## Conclusions

This study investigated the levels and factors of workplace stress, support for healthier lifestyles, and the use of stress-coping strategies among healthcare professionals in Ethiopia. The results illuminated a spectrum of workplace stress levels, with approximately 53% of participants reporting stress related to their workplace. Noteworthy socio-demographics factors influencing stress included gender, age, income status, and healthcare setting and types. While many healthcare workers reported experiencing better workplace support, a substantial number still perceived their support system as inadequate for healthier life style. This study highlights a gap in effectively supporting healthcare workers’ mental well-being and fostering healthier lifestyles within their work environments. Influenced by systemic challenges such as limited institutional support, variations in facility types, and income disparities, the findings indicate that stress management practices among frontline health workers remain suboptimal, with many not consistently engaging in key strategies such as regular exercise, relaxation techniques, and maintaining a healthy work-life balance.

### Implications and recommendations

This study underscores that healthcare workers in Ethiopia experience significant workplace stress driven by organizational, systemic, and socio-demographic factors, including workload, limited institutional support, income disparities, and facility-level differences. These stressors compromise staff well-being, job satisfaction, and retention, highlighting the need for targeted interventions. The findings indicate that current stress management practices are suboptimal, with many healthcare workers not consistently engaging in strategies such as regular exercise, relaxation techniques, and maintaining work-life balance. To address these challenges, we recommend implementing regular institutional stress management workshops and training programs focusing on time management, problem-solving skills, and coping strategies. Tailored psychosocial support, including counseling services, peer support groups, and educational programs sensitive to age, gender, income level, and facility type, should be provided to staff. Hospital managers should be trained to recognize workplace stress and foster supportive leadership practices, optimize workload distribution, strengthen communication channels, and provide mentorship and recognition programs. At a broader level, collaboration between hospital administrators, health policymakers, and regional health offices is essential to develop context-specific policies, staffing protocols, and implement tiered support programs for vulnerable staff, including younger and lower-income employees. Integration of pandemic-related and occupational stress management resources will further strengthen workplace resilience, contributing to staff well-being, retention, and more supportive and resilient local healthcare system.

Future research on workplace stress among health workers in Ethiopia should consider longitudinal designs to capture changes in stress levels over time and to evaluate the long-term effects of context-specific interventions. Interventional studies are also warranted to assess the effectiveness of stress-reduction frameworks within healthcare settings. Moreover, investment in capacity development, cross-sectoral collaboration, and the strengthening of primary healthcare facilities will be essential to support sustainable strategies for reducing workplace stress and to contribute to broader health systems strengthening.

## Supporting information

S1 Appendix Workplace Support for Health (WSHS) Scale. Shows the items used to measure perceived workplace support for healthy lifestyles, wellness leadership, and mental health resources.
(DOCX)

S2 Appendix Oslo Social Support Scale (OSSS-3). Shows the items used to assess perceived social support by gender, including support networks, concern from others, and practical help.
(DOCX)

S3 Appendix Stress Management Assessment Scale. Shows the items used to evaluate stress management practices, including time management, exercise, relaxation, mentoring, and problem-solving.
(DOCX)

S4 Appendix Supporting Data. Contains the dataset used for the study analyses, enabling transparency and reproducibility.
(XLS)

## References

[1] National Institute for Occupational Safety and Health (NIOSH). Stress . . . at work. 99–101. DHHS (NIOSH). 1999. https://www.cdc.gov/niosh/docs/99-101/default.html

[2] Giga SI, Hoel H, Lewis D. The cost of work-related stress to society: a systematic review. Br J Hosp Med (Lond). 2008;69(11):600–4.

[3] Stress statistics and facts. https://www.crossrivertherapy.com/stress-statistics-and-facts. Accessed 2023 October 1.

[4] EU-OSHA — European Agency for Safety and Health at Work. Economic impact of occupational safety and health in the member states of the European Union. Luxembourg: European Communities. 1999. https://osha.europa.eu/en/publications/reports/302

[5] National Center for Biotechnology Information. Stress at work. https://www.ncbi.nlm.nih.gov/books/NBK540859/

[6] Khan N, Khurshid S. Workplace stress and employee wellbeing: case of health care staff in UAE. ESJ. 2017;13(5):217. doi: 10.19044/esj.2017.v13n5p217

[7] Andrysiewicz T, Zygoń P. Workplace stress and employee wellbeing: case of healthcare staff in UA. J Econ Manag. 2019;36(2):45–56.

[8] Girma B, Nigussie J, Molla A, Mareg M. Occupational stress and associated factors among health care professionals in Ethiopia: a systematic review and meta-analysis. BMC Public Health. 2021;21(1):539. doi: 10.1186/s12889-021-10579-1 33740920 PMC7980550

[9] Ahmed F, Hawulte B, Yuya M, Birhanu S, Oljira L. Prevalence of burnout and associated factors among health professionals working in public health facilities of Dire Dawa city administration, Eastern Ethiopia. Front Public Health. 2022;10:836654. doi: 10.3389/fpubh.2022.836654 36033755 PMC9403244

[10] Efa AG, Lombebo AA, Nuriye S, Facha W. Prevalence of burnout and associated factors among nurses working in public hospitals, southern Ethiopia: a multi-center embedded mixed study. Sci Rep. 2024;14(1):31268. doi: 10.1038/s41598-024-82703-1 39732798 PMC11682220

[11] Werke EB, Weret ZS. Occupational stress and associated factors among nurses working at public hospitals of Addis Ababa, Ethiopia, 2022; a hospital-based cross-sectional study. Front Public Health. 2023;11:1147086. doi: 10.3389/fpubh.2023.1147086 37143975 PMC10151523

[12] Azumah C, Osei-Kufuor J, Oppong-Damoah R, Owusu B, Ohene-Asare J, Addo Yobo R. Perceived stress and its associated factors among healthcare workers in Gedeo zone, southern Ethiopia: a cross-sectional study. BMC Research Notes. 2021;14(1):1–6.33407799

[13] Hagos G, Tegegne A, Addis Z. Determinants of stress among healthcare workers: a systematic review and meta-analysis. Int J Environ Res Public Health. 2021;18(18):9260.34501849

[14] Centers for Disease Control and Prevention CDC. Workplace health promotion: the workplace health model. https://www.cdc.gov/workplacehealthpromotion/model/index.html. Accessed 2023 October 1.

[15] Federal Ministry of Health (FMoH). Health Sector Transformation Plan II (HSTP II). https://www.globalfinancingfacility.org/sites/default/files/Ethiopia-HSTP-II.pdf

[16] World Health Organization WHO. Mental health in the workplace: information sheet. Geneva: WHO. 2021. https://iris.who.int/bitstream/handle/10665/42625/9241590475.pdf

[17] Girma B, Nigussie J, Molla A, Mareg M. Occupational stress and associated factors among health care professionals in Ethiopia: a systematic review and meta-analysis. BMC Public Health. 2021;21(1):539. doi: 10.1186/s12889-021-10579-1 33740920 PMC7980550

[18] Joshi K, Modi B, Singhal S, Gupta S. Occupational stress among health care workers. Identifying Occupational Stress and Coping Strategies. IntechOpen. 2023. doi: 10.5772/intechopen.107397

[19] Mengist B, Amha H, Ayenew T, Gedfew M, Akalu TY, Assemie MA, et al. Occupational stress and burnout among health care workers in Ethiopia: a systematic review and meta-analysis. Arch Rehabil Res Clin Transl. 2021;3(2):100125. doi: 10.1016/j.arrct.2021.100125 34179761 PMC8212011

[20] Teshome A, Anteneh A, Misganaw E, Amare A, Endris M, Tsegaye B. Perceived stress and its associated factors among healthcare workers in Gedeo zone, southern Ethiopia: a cross-sectional study. BMC Res Notes. 2021;14(1):1–6.33407799

[21] Kava CM, Passey D, Harris JR, Chan KCG, Hannon PA. The workplace support for health scale: reliability and validity of a brief scale to measure employee perceptions of wellness. Am J Health Promot. 2021;35(2):179–85. doi: 10.1177/0890117120949807 32808553 PMC7870498

[22] Chen L, Hannon PA, Laing SS, Kohn MJ, Clark K, Pritchard S, et al. Perceived workplace health support is associated with employee productivity. Am J Health Promot. 2015;29(3):139–46. doi: 10.4278/ajhp.131216-QUAN-645 25559250

[23] Kocalevent R-D, Berg L, Beutel ME, Hinz A, Zenger M, Härter M, et al. Social support in the general population: standardization of the Oslo social support scale (OSSS-3). BMC Psychol. 2018;6(1):31. doi: 10.1186/s40359-018-0249-9 30016997 PMC6050647

[24] Bøen H, Dalgard OS, Bjertness E. The importance of social support in the associations between psychological distress and somatic health problems and socio-economic factors among older adults living at home: a cross sectional study. BMC Geriatr. 2012;12:27. doi: 10.1186/1471-2318-12-27 22682023 PMC3464708

[25] Jayaraman T. Stress Management Questionnaire. https://www.scribd.com/doc/55025282/Stress-Management-Questionnaire. Accessed 2025 December 14.

[26] Lazarus RS, Folkman S. Stress coping skill-10 items. Psychol Stress Coping. 1984;1(1):14–8

[27] de Oliveira C, Saka M, Bone L, Jacobs R. The Role of Mental Health on Workplace Productivity: A Critical Review of the Literature. Appl Health Econ Health Policy. 2023;21(2):167–93. doi: 10.1007/s40258-022-00761-w 36376610 PMC9663290

[28] Hall LH, Johnson J, Watt I, Tsipa A, O’Connor DB. Healthcare Staff Wellbeing, Burnout, and Patient Safety: A Systematic Review. PLoS One. 2016;11(7):e0159015. doi: 10.1371/journal.pone.0159015 27391946 PMC4938539

[29] West CP, Dyrbye LN, Erwin PJ, Shanafelt TD. Interventions to prevent and reduce physician burnout: a systematic review and meta-analysis. Lancet. 2016;388(10057):2272–81. 27692469 10.1016/S0140-6736(16)31279-X

[30] Dall’Ora C, Ball J, Griffiths P. The global burden of nursing shift work: a systematic review. J Nurs Scholarsh. 2022;54(1):12–22.

[31] Aberhe W, Mariye T, Bahrey D, Hailay A, Mebrahtom G, Zereabruk K, et al. Job stress among nurses in Ethiopia: A systematic review and meta-analysis. International J Africa Nursing Sci. 2024;20:100661. doi: 10.1016/j.ijans.2024.100661

[32] Bakare M, Darega J, Nugus GG, Tsegaw M. Work-related stress and associated factors among health professionals working in Ambo town public health facilities, West Shoa Zone, Ethiopia, 2021: a cross-sectional study. 2023. Available from: https://bmjopen.bmj.com/content/13/11/e074946 10.1136/bmjopen-2023-074946 PMC10679989 38000820

[33] Yesuf SM, Derseh BT, Girma D, Dejene TM. Work-related stress and associated factors among health professionals in zone 1, Afar region, Ethiopia. Heliyon. 2022;8(12):e12167. doi: 10.1016/j.heliyon.2022.e12167 PMC9755361 36531619

[34] Belachew T, Birhanu Z. Occupational stress and its associated factors among health care professionals in Ethiopia: a systematic review and meta-analysis. J Environ Public Health. 2018;2018:6286010. https://www.hindawi.com/journals/jeph/2018/6286010/30598668

[35] Bakker AB, Demerouti E. The job demands-resources model: state of the art. J Manag Psychol. 2007;22(3):309–28.

[36] Halbesleben JRB. Sources of social support and burnout: a meta-analytic test of the conservation of resources model. J Appl Psychol. 2006;91(5):1134–45. doi: 10.1037/0021-9010.91.5.1134 16953774

[37] Health Promotion and Disease Prevention Directorate (HPDP). Improving employee health in the workplace. https://hpdp.gov.mt/sites/default/files/2023-08/improving_employee_health_in_the_workplace_en.pdf. 2023. Accessed 2025 July 11.

[38] Labrague LJ, McEnroe-Petitte DM, Leocadio MC, Van Bogaert P, Cummings GG. Stress and ways of coping among nurse managers: a systematic review. J Nurs Manag. 2017;25(3):147–57.

[39] Hakanen JJ, Bakker AB, Schaufeli WB. Burnout and work engagement among teachers. J Sch Psychol. 2006;43(6):495–513.

[40] Stults-Kolehmainen M, Sinha R. The effects of stress on physical activity and exercise. Health Psychol Rev. 2014;8(2):182–97. 10.1007/s40279-013-0090-5 PMC3894304 24030837

[41] Greenhaus JH, Allen TD. Work–family balance: a review and extension of the literature. In: Anderson N, Ones DS, Sinangil HK, Viswesvaran C, editors. Handbook of Industrial, Work & Organizational Psychology. London: Sage Publications. 2011. p. 165–83.

[42] Girma B, Nigussie J, Molla A, Mareg M. Occupational stress and associated factors among health care professionals in Ethiopia: a systematic review and meta-analysis. BMC Public Health. 2021;21(1):539. doi: 10.1186/s12889-021-10579-1 33740920 PMC7980550

[43] Odonkor ST, Adams S. Predictors of stress and associated factors among healthcare workers in Western Ghana. Heliyon. 2021;7(6):e07223. doi: 10.1016/j.heliyon.2021.e07223 34159275 PMC8203702

[44] Lantz PM, House JS, Mero RP, Williams DR. Stress, life events, and socioeconomic disparities in health: results from the Americans’ Changing Lives Study. J Health Soc Behav. 2005;46(3):274–88. doi: 10.1177/002214650504600305 16259149

[45] World Health Organization WHO. Occupational health: stress at the workplace. https://www.ncbi.nlm.nih.gov/pmc/articles/PMC9755361/pdf/main.pdf. Accessed 2025 July 11.

[46] Habtu Y, Kumie A, Selamu M, Harada H, Girma E. Prevalence and determinants of occupational depression, anxiety, and stress among Ethiopian healthcare workers. Sci Rep. 2024;14(1):21817. doi: 10.1038/s41598-024-72930-x 39294429 PMC11410813

[47] Nyirigira G, Bailey JG, Rutayisire F, Neil KL, Bould MD, Kwizera R, et al. Staff burnout and its risk factors at King Faisal Hospital Rwanda: a cross-sectional survey. BMC Health Serv Res. 2025;25(1):508. doi: 10.1186/s12913-025-12638-4 40197340 PMC11977868

[48] Lwiza AF, Lugazia ER. Burnout and associated factors among healthcare workers in acute care settings at a tertiary teaching hospital in Tanzania: An analytical cross-sectional study. Health Sci Rep. 2023;6(5):e1256. doi: 10.1002/hsr2.1256 37152234 PMC10160764

[49] American Psychological Association. Work, stress, and health. https://www.apa.org/pi/ses/resources/publications/work-stress-health. Accessed 2025 July 11.

[50] Poncet MC, Chiron C, Boudouin D, Dube L, Constantin JM, Mira JP. Burnout syndrome in critical care nursing: a review of the literature. Am J Crit Care. 2007;16(3):235–41.

[51] McGowan J. The role of organizational culture in employee well-being. Int J Organ Anal. 2016;24(4):704–16.

[52] Ganster DC, Rosen CC. Work stress and employee health: a multidisciplinary review. J Manag. 2013;39(5):1085–122. https://www.sciencedirect.com/science/article/abs/pii/S000187919891661X

