# Correction: Workplace stress, support and stress management strategies for healthier lifestyles among healthcare workers in Ethiopia

**DOI:** 10.1371/journal.pone.0345094

**Published:** 2026-03-16

**Authors:** Zewdie Birhanu, Habtamu Mekonnen, Reginald A. Kavishe, Lars Lien, Lena Skovgaard Andersen, Declare L. Mushi, Tania Aase Dræbel, Gudina Terefe Tucho

The second author’s name is spelled incorrectly. The correct name is: Habtamu Mekonnen. The correct citation is: Birhanu Z, Mekonnen H, Kavishe RA, Lien L, Andersen LS, Mushi DL, et al. (2026) Workplace stress, support and stress management strategies for healthier lifestyles among healthcare workers in Ethiopia. PLoS One 21(1): e0341226. https://doi.org/10.1371/journal.pone.0341226
