# Supplementary material for: Workplace stress, support and stress management strategies for healthier lifestyles among healthcare workers in Ethiopia
Source: PLoS One. 2026 Jan 29;21(1):e0341226. doi: 10.1371/journal.pone.0341226 (PMC12854458; doi:10.1371/journal.pone.0341226)
Supplement: S1 Appendix — Shows the items used to measure perceived workplace support for healthy lifestyles, wellness leadership, and mental health resources. (DOCX) [file pone.0341226.s001.docx]

**Appendix S1: Workplace Support for Health (WSHS)**

| Items | Strongly agree | Agree | Neutral/undecided | Disagree | Strongly disagree |
| --- | --- | --- | --- | --- | --- |
| 1. Overall, my **workplace** supports me in living a healthier life. |  |  |  |  |  |
| 1. My **supervisor** supports me in living a healthier life. |  |  |  |  |  |
| 1. Most employees here have healthy habits. |  |  |  |  |  |
| 1. At my workplace we have one or more leaders (e.g. CEOs or managers) who are wellness champions. |  |  |  |  |  |
| 1. At my workplace we have one or more employees who are wellness champions. |  |  |  |  |  |
| 1. At my workplace there is support for health workers who experience some mental health problems |  |  |  |  |  |
| 1. At my workplace there resources ( guides, information, aids) on mental health |  |  |  |  |  |
