# Supplementary material for: Workplace stress, support and stress management strategies for healthier lifestyles among healthcare workers in Ethiopia
Source: PLoS One. 2026 Jan 29;21(1):e0341226. doi: 10.1371/journal.pone.0341226 (PMC12854458; doi:10.1371/journal.pone.0341226)
Supplement: S2 Appendix — Shows the items used to assess perceived social support by gender, including support networks, concern from others, and practical help. (DOCX) [file pone.0341226.s002.docx]

**Appendix S2**

***Oslo Social Support Scale (OSSS-3):*** Perceived social support by gender based on the SSS-3 classification scale

1. **How many people can you count on if you have severe personal problems?**

- None
- One or two
- Three to five
- More than five

1. **How much concern do people show in what you are doing?**

- No concern or interest
- Uncertain
- Some concern and interest
- A lot of concern and interest

1. **How easy is it to get practical help from neighbors?**

- Very difficult
- Difficult
- Easy
- Very easy

**Appendix S3: Stress management assessment scale**

| When I faced with stressful situation | Strongly agree | Agree | Neutral/  undecided | Disagree | Strongly disagree |
| --- | --- | --- | --- | --- | --- |
| 1. I use effective time-management methods such as keeping track of my time, making to do lists, and prioritizing tasks. |  |  |  |  |  |
| 1. I maintain a program of regular exercise for fitness. |  |  |  |  |  |
| 1. I maintain an open, trusting relationship with someone with whom I can share my frustrations. |  |  |  |  |  |
| 1. I know and practice several temporary relaxation techniques such as deep breathing and muscle relaxation. |  |  |  |  |  |
| 1. I frequently affirm my priorities so that less important things don’t drive out more important things. |  |  |  |  |  |
| 1. I maintain balance in my life by pursuing a variety of interests outside of work. |  |  |  |  |  |
| 1. I have a close relationship with someone who serves as my mentor or advisor. |  |  |  |  |  |
| 1. I effectively utilize others in accomplishing work assignments. |  |  |  |  |  |
| 1. I encourage others to generate recommended solutions, not just questions, when they come to me with problems or issues. |  |  |  |  |  |
| 1. I strive to redefine problems as opportunities for improvement. |  |  |  |  |  |
