# Supplementary material for: Workplace stress, support and stress management strategies for healthier lifestyles among healthcare workers in Ethiopia
Source: PLoS One. 2026 Jan 29;21(1):e0341226. doi: 10.1371/journal.pone.0341226 (PMC12854458; doi:10.1371/journal.pone.0341226)
Supplement: S3 Appendix — Shows the items used to evaluate stress management practices, including time management, exercise, relaxation, mentoring, and problem-solving. (DOCX) [file pone.0341226.s003.docx]

**Appendix S3: Stress management assessment scale**

| When I faced with stressful situation | Strongly agree | Agree | Neutral/  undecided | Disagree | Strongly disagree |
| --- | --- | --- | --- | --- | --- |
| 1. I use effective time-management methods such as keeping track of my time, making to do lists, and prioritizing tasks. |  |  |  |  |  |
| 1. I maintain a program of regular exercise for fitness. |  |  |  |  |  |
| 1. I maintain an open, trusting relationship with someone with whom I can share my frustrations. |  |  |  |  |  |
| 1. I know and practice several temporary relaxation techniques such as deep breathing and muscle relaxation. |  |  |  |  |  |
| 1. I frequently affirm my priorities so that less important things don’t drive out more important things. |  |  |  |  |  |
| 1. I maintain balance in my life by pursuing a variety of interests outside of work. |  |  |  |  |  |
| 1. I have a close relationship with someone who serves as my mentor or advisor. |  |  |  |  |  |
| 1. I effectively utilize others in accomplishing work assignments. |  |  |  |  |  |
| 1. I encourage others to generate recommended solutions, not just questions, when they come to me with problems or issues. |  |  |  |  |  |
| 1. I strive to redefine problems as opportunities for improvement. |  |  |  |  |  |
